# Influence of Nb Content on Precipitation, Grain Microstructure, Texture and Magnetic Properties of Grain-Oriented Silicon Steel

**DOI:** 10.3390/ma13235581

**Published:** 2020-12-07

**Authors:** Yong Wang, Chengyi Zhu, Guangqiang Li, Yulong Liu, Yu Liu

**Affiliations:** 1 State Key Laboratory of Refractories and Metallurgy, Wuhan University of Science and Technology, Wuhan 430081, China; wangyong0911@wust.edu.cn (Y.W.); zhchyhsy@wust.edu.cn (C.Z.); liuyulong@wust.edu.cn (Y.L.); liuyu629@wust.edu.cn (Y.L.); 2 Key Laboratory for Ferrous Metallurgy and Resources Utilization of Ministry of Education, Wuhan University of Science and Technology, Wuhan 430081, China; 3 Collaborative Innovation Center for Advanced Steels, Wuhan University of Science and Technology, Wuhan 430081, China

**Keywords:** niobium, grain-oriented silicon steel, precipitates, microstructure, texture, magnetic properties

## Abstract

The effects of Nb content on precipitation, microstructure, texture and magnetic properties of primary recrystallized grain-oriented silicon steel were investigated by various methods. The results show that the precipitates in primary recrystallized sheets are mainly MnS, Nb(C,N), composite precipitates of MnS and AlN, and composite precipitates of Nb(C,N) and AlN. Adding niobium could refine the primary recrystallized microstructure. The steel with 0.009 wt% Nb possesses the finest and the most dispersed precipitates, which contributes to the finest primary recrystallized microstructure due to the strong pinning force. Adding niobium is beneficial to obtain large volume fraction favorable texture for grain-oriented silicon steel, and the effect of Nb addition is not obvious when the content is higher than 0.009 wt%. After final annealing, the steel with 0.009 wt% Nb shows the best magnetic properties, *B*_800_ = 1.872 T, *P*_1.7/50_ = 1.25 W/kg.

## 1. Introduction

Since the grain-oriented silicon steel was proposed in 1934 by Goss [1], the steel has been used in electricity and electronic devices for nearly one hundred years [2]. Its excellent magnetic properties are related to the sharpness of Goss texture ({110}<001>), which is mainly obtained by secondary recrystallization. From the steel melting to the final product, the grain-oriented silicon steel undergoes a series of metallurgical process such as steelmaking, continuous casting, slab reheating and hot rolling, normalizing annealing, and cold rolling. Moreover, decarburization annealing, nitriding and secondary recrystallization annealing were conducted to obtain sharp Goss texture [3]. During the past several decades, many investigations on optimizing magnetic properties have been carried out, such as increasing Si content [4,5,6], reducing final product thickness [7,8], or others. However, high silicon content in steel causes the formation of ordered phases, which results in extremely poor formability at room temperature [9]. As regards reducing final product thickness, it will consume more energy to reduce the final thickness. In addition, the magnetic induction will decrease rapidly due to the lack of secondary Goss nuclei and the increase of grains with other orientation caused by high reduction ratio, which have a competitive relationship with Goss grains during abnormal growth process [10].

It is well known that the inhibitors play an important role during the production of grain-oriented silicon steel. It prevents the normal growth of the primary recrystallized grains and promotes the abnormal growth of Goss grains during secondary recrystallization annealing as the temperature arrives at a certain value. At present, many types of inhibitors have been used for controlling sharp Goss texture in grain-oriented silicon steel, such as AlN [11], MnS [12], and Cu_2−x_S [13]. The slab is reheated at high temperature before hot rolling, and the coarse particles in the slab are redissolved into the matrix; after that, fine and dispersed inhibitors are precipitated during hot rolling process. In order to dissolve the precipitates into the matrix completely, the slabs are usually reheated above 1350 °C, which inevitably brings about a series of problems, such as energy waste, low yield, and high cost [14,15]. Therefore, it is essential to develop new inhibitors with low solution temperature, which has attracted extensive attentions in recent years. Many researches show that niobium used as an assistant inhibitor formation element to form Nb(C, N) is feasible. Feng’s results show that Nb(C,N) is obtained after Nb is added into silicon steel, and it makes little effect on the precipitation of MnS; moreover, the slab reheating temperature can be reduced with proper Nb content in silicon steel, but it has a negative effect with excessive Nb addition because that the inhibitors cannot dissolve into the matrix totally at high temperature [16,17]. Klaus Hulka et al. report that the hot strip material of niobium microalloyed steel contains the highest volume fraction of Goss texture and that the core losses are the lowest in the sheet with NbC as main inhibitor [18]. Furthermore, it has been confirmed that the grain-oriented silicon steel with niobium could experience complete abnormal growth during high temperature annealing, which shows a better magnetic induction (*B*_8_ was 1.88 T) [19]. Our previous study [20] showed that the precipitates in the hot-rolled steel with 0.028 wt% Nb are finer and more dispersed, than in the steel with higher Nb content. This leads to smaller grain size and higher intensity of Goss texture owing to its strong pinning force. However, the Al_s_ content in the previous study was really low, which led to poor magnetic properties after secondary recrystallization annealing. In addition, the state of primary recrystallized has an important effect on secondary recrystallization, which affects the magnetic properties of the final product. However, there are few researches on the influence of Nb on the formation of primary recrystallization, which need further studied.

In the current study, the composition of silicon steel is optimized; the Al_s_ content increases to 0.024 wt%, and Nb content decreases below 0.028 wt%. Four slabs with different Nb content were produced by hot rolling, normalizing annealing, cold rolling, primary recrystallization annealing, nitriding, and secondary recrystallization annealing. The influence of Nb on precipitates, microstructure, and texture of primary recrystallized grain-oriented silicon steel was studied in detail. The purpose of this work was to provide the fundamental knowledge for the production of Nb-bearing grain-oriented silicon steel.

## 2. Materials and Methods

Four grain-oriented silicon steels with different Nb content were prepared by a 100 kg vacuum induction furnace ((Jinzhou Electric Furnace Co., Ltd., Jinzhou, China) and were forged to 210 × 120 × (30–50) mm square ingot. The main chemical composition of the specimens is listed in Table 1. The ingots were reheated to 1180 °C for 90 min, and then were hot rolled to 2.3 mm in thickness. Then the hot-rolled bands were treated by two-stage normalizing annealing. The hot-rolled bands were heated to 1140 °C and held for 50 s, and then the bands were rapidly cooled to 930 °C and held for 120 s, and subsequently cooled down in boiling water. Following two-stage normalizing annealing, the bands were cold rolled to 0.23 mm in thickness, the corresponding reduction rate is 90%. The cold rolled sheets were then primarily annealed at 840 °C for 200 s under a wet atmosphere which comprised 45% H_2_ and 55% N_2_ (volume fraction), and *P*_H2O_:*P*_H2_ = 0.27. Subsequently, the primary annealed sheets were subjected to nitriding for 60 s at 800 °C. Finally, the bands were heated to 1200 °C at a heating rate of 20 °C/h under 75% H_2_ and 25% N_2_ and lasted for 10 h under pure H_2_ for purification.

**Figure 1:**
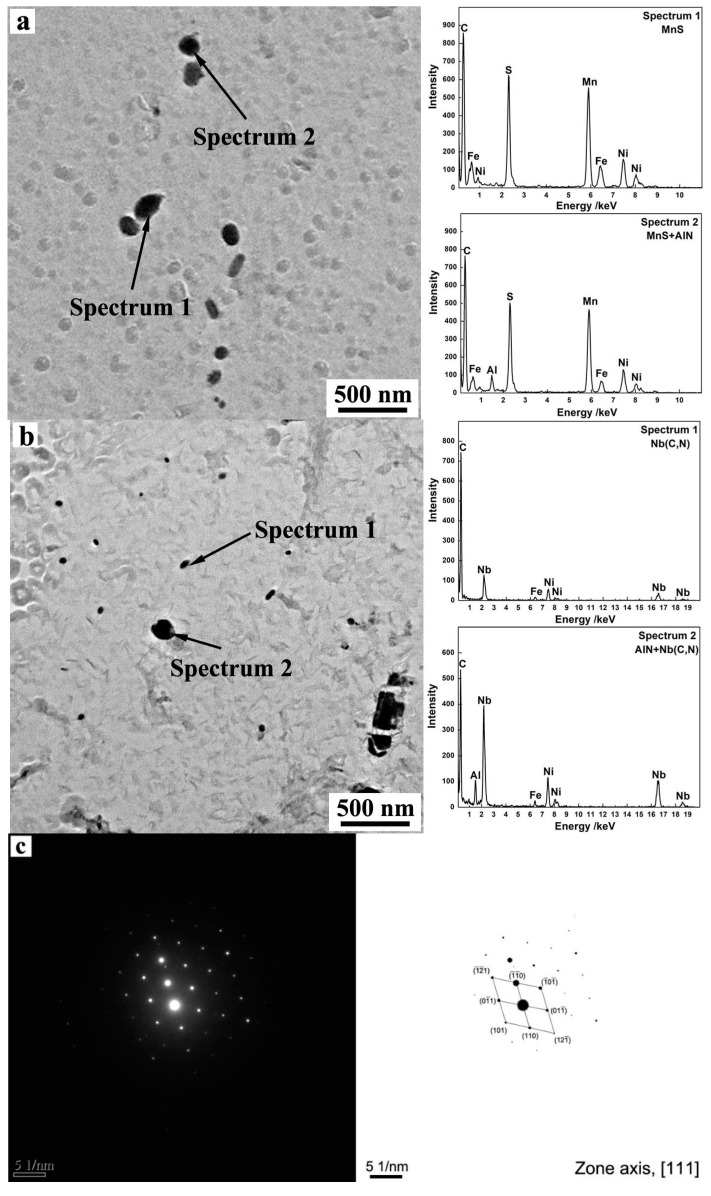
Typical shape and EDS analysis of precipitates in primary recrystallized sheet. (**a**) Precipitates in S1, (**b**) precipitates in S3, (**c**) electron diffraction patterns of Spectrum 1 in Figure 1b.

**Figure 2:**
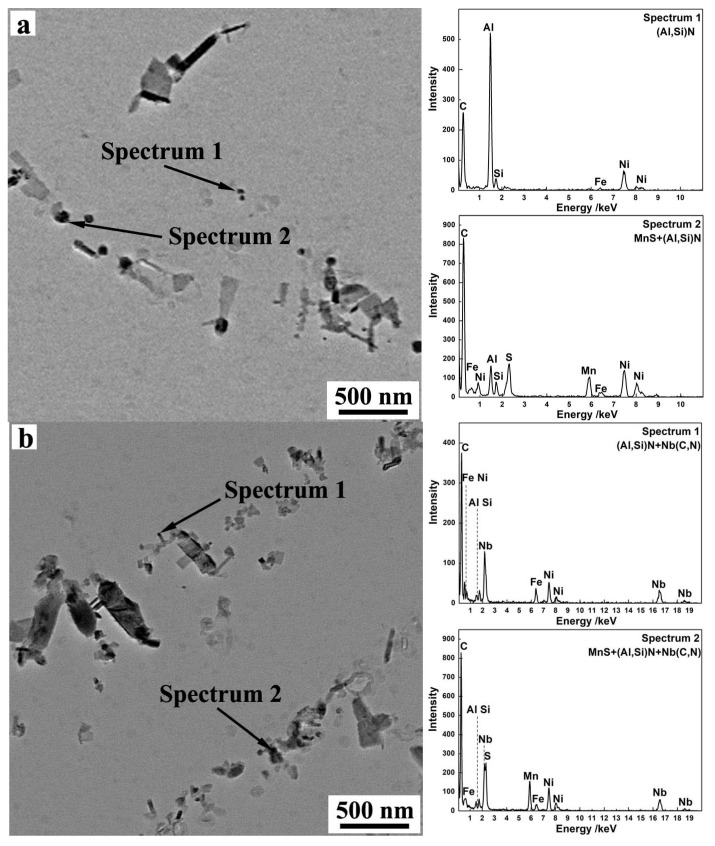
Typical shape and EDS analysis of precipitates in nitrided sheet: (**a**) precipitates in S1, (**b**) precipitates in S3.

In this study, the characterization of precipitates and microstructure and texture of primary recrystallized grain-oriented silicon steel were investigated in detail. The precipitates of decarburized bands were extracted by carbon extraction replica technique and analyzed by JEM-2100 transmission electron microscopy (TEM) (Joel, Tokyo, Japan) equipped with energy dispersive X-ray spectroscopy (EDS). One hundred TEM images were taken for each specimen, and the Image-Pro Plus image analysis software were used for measuring the areal density and size of the precipitates. Previous studies show that the inhibitors with size in the range of 20–100 nm are effective and process strong ability of inhibiting the grain growth [21], so only the precipitates with size less than 100 nm were investigated. In addition, in the process of preparing carbon replica, carbon replica was firstly immersed into water to separate from a steel matrix and then was salvaged by Ni net and dried. The replica was observed with Ni net together under the electron beam. Therefore, strong peaks from Ni can be observed in Figure 1 and Figure 2.

The microstructure and texture along the longitudinal section (RD-ND) of decarburized bands were determined by electron backscattered diffraction (EBSD) system attached to a field emission scanning electron microscope (SEM, Apreo S Hivac) (FEI Company, Hillsboro, CA, USA). The EBSD data was analyzed by an HKL Channel 5 EBSD software (Oxford Instruments, Oxford, UK) and presented in the form of orientation map and orientation distribution functions (ODF) in φ2 = 45° section. Finally, the magnetic inductions at 800 A/m (*B*_800_) and the core loss at 1.7 T and 50 Hz (*P*_1.7/50_) were measured by soft magnetic measuring instrument MPG-100D (Brockhaus, Leipzig, Germany).

## 3. Results and Discussion

### 3.1. Characterization of Precipitates in Steels

Figure 1 shows the precipitates in primary recrystallized sheets. It should be noticed that a strong carbon peak is obtained in each image because that the carbon replica specimens for precipitates observation were examined by TEM. Meanwhile, nitrogen is a kind of light element, the peaks of N in nano-scaled precipitates are difficult to be detected by EDS method. The precipitates in S1 are mainly MnS and composite precipitates of MnS and AlN. It is obvious that MnS is spherical and the composite precipitate of MnS and AlN is nearly ellipsoidal shaped. After adding niobium, fine precipitates were detected to be Nb(C,N), as shown in Figure 1b. In addition, the composite precipitates of Nb(C,N) and AlN were also observed in S3. Figure 1c shows the electron diffraction patterns of Spectrum 1 in Figure 1b, it indicates that the precipitate is fcc-Nb(C,N).

Figure 2 shows the precipitates in nitrided sheets. Obviously, the precipitates become finer and more disperse. Many fine (Al,Si)N precipitates formed after nitriding. The precipitates with larger size were detected to be composite precipitates of MnS and (Al,Si)N, composite precipitates of (Al,Si)N and Nb(C,N), composite precipitates of MnS, (Al,Si)N and Nb(C,N). It is interesting to find that only the composite precipitates containing MnS show a large size, while the composite precipitates containing Nb(C,N) show a small size, which indicates that niobium could refine the size of precipitates.

**Figure 3:**
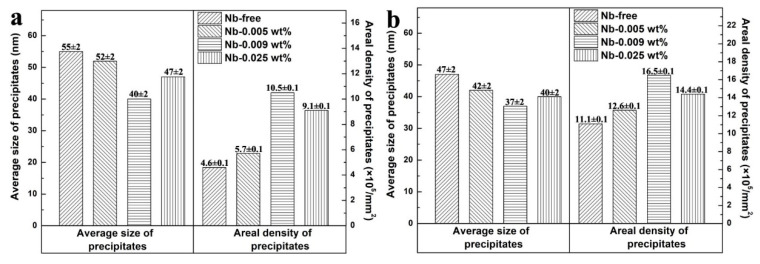
Average size and areal density of precipitates in (**a**) primary recrystallized sheet and (**b**) nitrided sheet.

Furthermore, in order to investigate the effect of Nb content on precipitates, the distribution and size of the precipitates of each sample were counted, and the statistical results are shown in Figure 3. According to Figure 3a, the average size of precipitates in S1 is 55 nm, the areal density of precipitates in S1 is 4.6 × 10^5^/mm^2^. As adding 0.005 wt% Nb, the average size of precipitates reduces to 52 nm, meanwhile the areal density of precipitates increases to 5.7 × 10^5^/mm^2^. With the continued addition of Nb, the finest and most dispersed precipitates are obtained in S3, the average size and areal density of precipitates are 40 nm and 10.5 × 10^5^/mm^2^, respectively. Comparing the precipitates in S3 and S4, the precipitates in S4 is larger and less dispersed. This result may be related to the distribution of previous precipitates. Our previous results show that the precipitates in normalized sheet become finer and more dispersed as the niobium content varying from 0 to 0.009 wt%. However, as the niobium increases to 0.025 wt%, the precipitates in normalized sheet are larger and less dispersed. After nitriding, the precipitates in each specimen become finer and more dispersed because of the formation of fine (Al,Si)N during nitriding, as shown in Figure 3b. It should be noticed that the average size and areal density of precipitates in S3 are still the smallest and the largest, respectively.

### 3.2. Effect of Nb on Microstructure of Primary Recrystallized Grain-Oriented Silicon Steel

**Figure 4:**
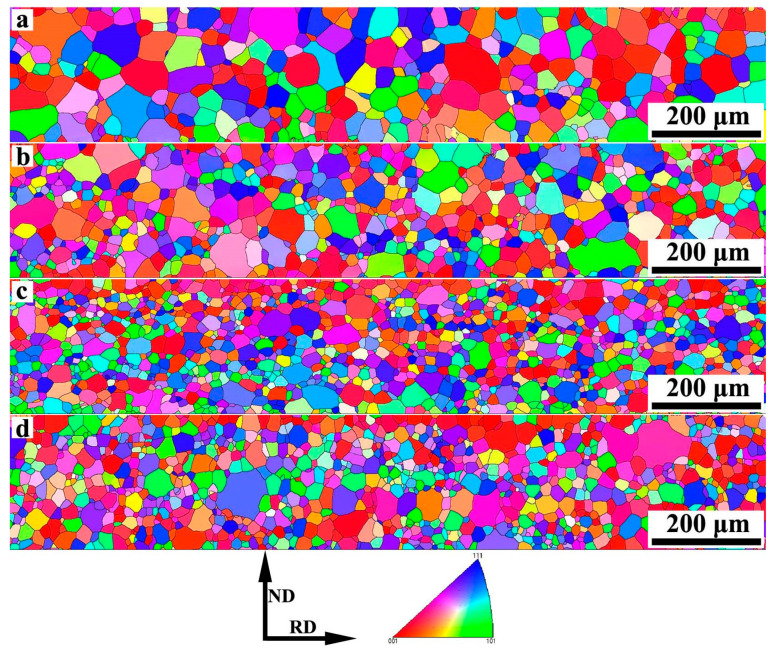
Inverse pole figure (IPF) of primary recrystallized sheets with different Nb content (**a**) Nb-free, (**b**) Nb-0.005 wt%, (**c**) Nb-0.009 wt%, (**d**) Nb-0.025 wt%.

**Figure 5:**
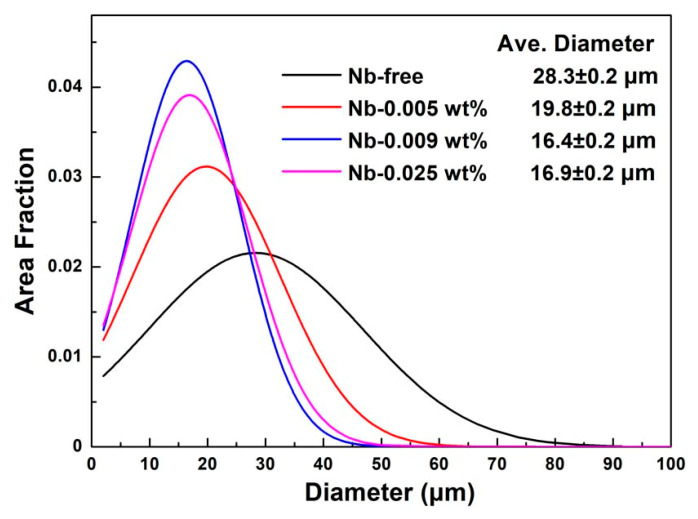
Grain diameter distribution after primary annealing.

**Figure 6:**
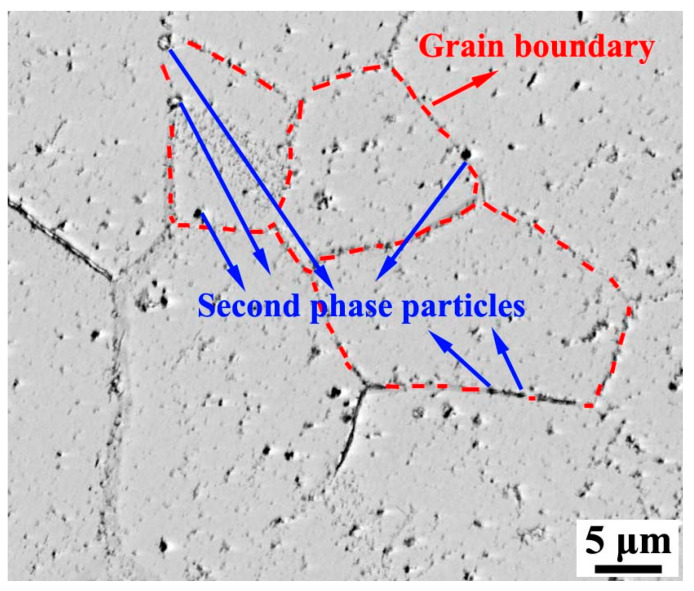
Second phase particle exist around the grain boundary of primary recrystallized steel with 0.009 wt% Nb.

Figure 4 shows the inverse pole figure (IPF) of primary recrystallized sheets with different Nb content. It is obvious that all of the samples are completely recrystallized and consist of equiaxed grains through the thickness. It is easy to find that the grains are refined after Nb is added, and the grain diameter distribution after primary annealing is shown in Figure 5. The average grain size in S1 is 28.3 μm, the grain size mainly distributes in the range of 20–40 μm. After adding 0.005 wt% Nb, the average grain size reduced to 19.8 μm, the grain size mainly distributes in the range of 15–30 μm. As increasing Nb content, it is interesting to find that the microstructure of S3 and S4 are similar. According to Figure 5, the microstructure of S3 is the finest, the grain size mainly distributes in the range of 10–25 μm. In this study, all the specimens are treated in an identical way, the size and distribution of precipitates could explain the difference of primary recrystallized microstructure. It is well known that the grain boundaries could be hindered by inhibitors when a grain boundary is moving through a matrix with second phase particles [22], as shown in Figure 6. The pinning force can be calculated by Equation (1) [23]:

(1) $$P_{z}=\frac{3f_{v}\cdot \gamma }{d}$$

where *P_z_* is the pining force of the inhibitors, *f_v_* is the volume fraction of the inhibitors, *γ* is the grain boundary energy, and *d* is the average diameter of the inhibitors. It can be concluded that the pining force increases with decreasing the size of inhibitors and increasing the distribution density. According to Figure 3, the precipitates in primary recrystallized sheet of S3 are finer and more dispersed. Considering that all specimens are treated in an identical way, it is reasonable to deduce that the pinning force of precipitates in cold-rolled sheet of S3 is the strongest. As a result, the grain growth is inhibited by precipitates during decarburization annealing process, and fine primary recrystallized microstructure is obtained in S3. Previous researches have proved that the Goss grains are easier to swallow fine and uniform primary grains surrounding them and become abnormal large size during final annealing process [24,25]. Therefore, adding niobium could refine the primary recrystallized microstructure, which could provide proper microstructure for final annealing. Meanwhile, it should be noticed that the difference between the primary recrystallized microstructure of S3 and S4 is really small, which indicates that adding 0.009 wt% Nb is enough for grain-oriented silicon steel.

### 3.3. Effect of Nb on Texture of Primary Recrystallized Grain-Oriented Silicon Steel

**Figure 7:**
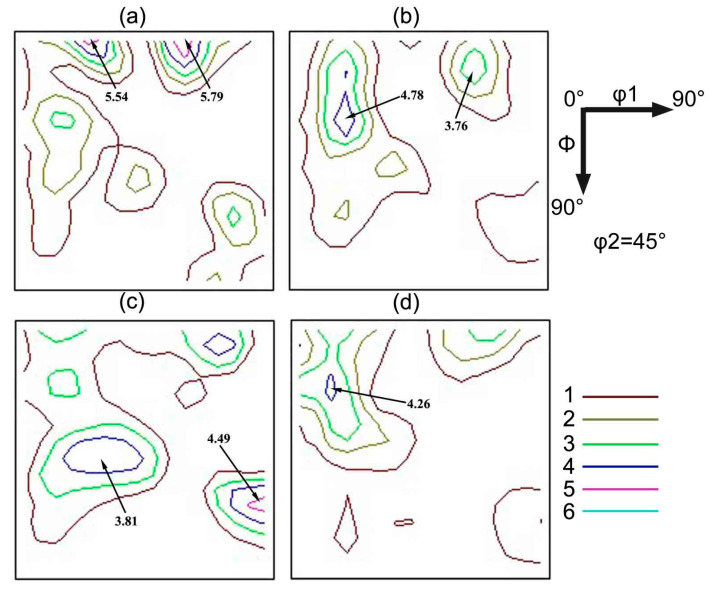
ODFs at φ2 = 45° of primary recrystallized sheet: (**a**) Nb-free, (**b**) Nb-0.005 wt%, (**c**) Nb-0.009 wt%, (**d**) Nb-0.025 wt%.

Figure 7 shows the φ2 = 45° sections of orientation distribution functions (ODF) of four primary recrystallized sheets. It can be observed that all the specimens possess the same texture type, which are α-fiber around {001}<110> and γ-fiber around {111}<112>. However, the intensity of texture is changed after adding niobium. The specimen without Nb shows strong α-fiber with peaks at {001}<110>, and the intensity of {001}<110> is 5.54, as increasing Nb content, α-fiber becomes weaker, and strong γ-fiber with peaks at {111}<112> is obtained in S3, the intensity of {111}<112> is 4.49. In this study, strong {001}<110> and γ-fiber would form in cold rolled sheets with a heavy reduction rate of low carbon steel [26]. During the decarburization annealing, the recrystallization nucleation rate and grain growth rate are related to the stored energy of cold rolled deformed grains. The stored energy of deformed grains is higher, the grain of which prefers to nucleate during annealing process. It is known that the stored energy of deformed grains in oriented silicon steel depends on grain orientation, which decreases in following order: *E*_{110}_ > *E*_{111}_ > *E*_{112}_ > *E*_{100}_ [27]. As mentioned earlier, the strongest pining force is obtained in S3, because of two aspects, the recrystallization nucleation rate and grain growth rate of α-fiber are severely inhibited, which shows strong γ-fiber with peaks at {111}<112> in S3.

**Figure 8:**
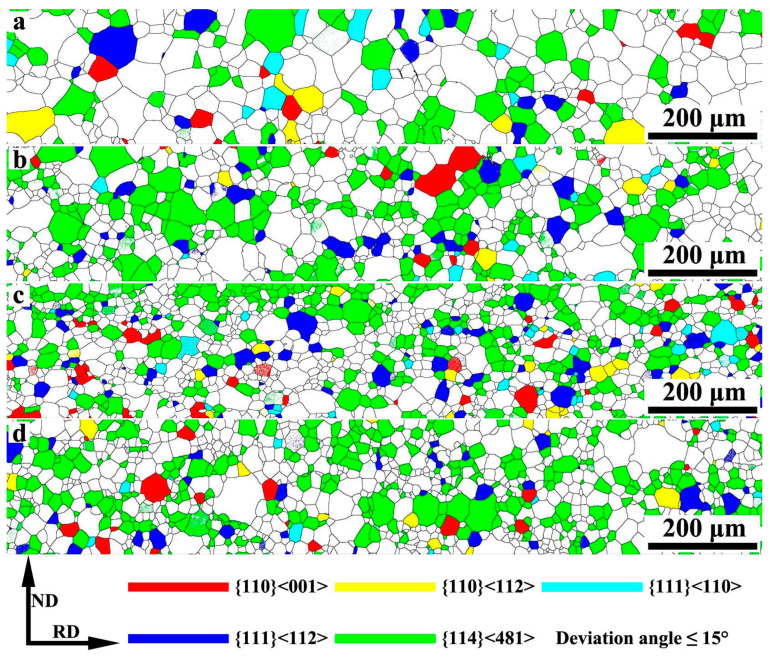
Orientation image maps (OIM) of several main components in primary recrystallized sheets: (**a**) Nb-free, (**b**) Nb-0.005 wt%, (**c**) Nb-0.009 wt%, (**d**) Nb-0.025 wt%.

Furthermore, in order to study the effect of Nb on texture of primary recrystallized sheets, the orientation image maps (OIM) of several main components in primary recrystallized sheets are shown in Figure 8. Table 2 gives the corresponding volume fraction of main texture in different specimens. It is reported that Brass texture ({110}<112>) could compete with Goss texture ({110}<001>) and grow up abnormally during final annealing, because both Brass and Goss texture belong to {110} type texture, and the both have obvious surface energy advantage on {110} planes in hydrogen atmosphere [28]. If the Brass texture abnormally grows up during final annealing, it will easily penetrate through the sheet and decrease magnetic properties. According to Figure 8 and Table 2, the volume fraction of Brass texture decreases with the increase of Nb content; it reduces from 3.39% to 1.55%. Therefore, adding niobium could decrease the volume fraction of unfavorable texture for grain-oriented silicon steel. Meanwhile, it can be seen that the volume fraction of Goss texture increases with the increase of Nb content, and it reaches the maximum value when adding 0.009 wt% Nb; the maximum value is 3.24%. Moreover, it is well known that γ-fiber and {114}<481> texture are related ∑9 with Goss texture, which are beneficial for Goss grains to swallow them and grow up abnormally during final annealing [29]. As increasing Nb content, the volume fraction of favorable texture increases obviously, especially for {111}<112> and {114}<481> texture. It should be noticed that the sum of {111}<112> and {114}<481> texture changes slightly between S3 and S4. Given the above, it is reasonable to deduce that adding niobium is beneficial to obtain larger volume fraction favorable texture for grain-oriented silicon steel, and the effect of Nb addition is not obvious when the content is higher than 0.009 wt%.

### 3.4. Effect of Nb on Macrostructure and Magnetic Properties of Secondary Annealed Sheet

**Figure 9:**
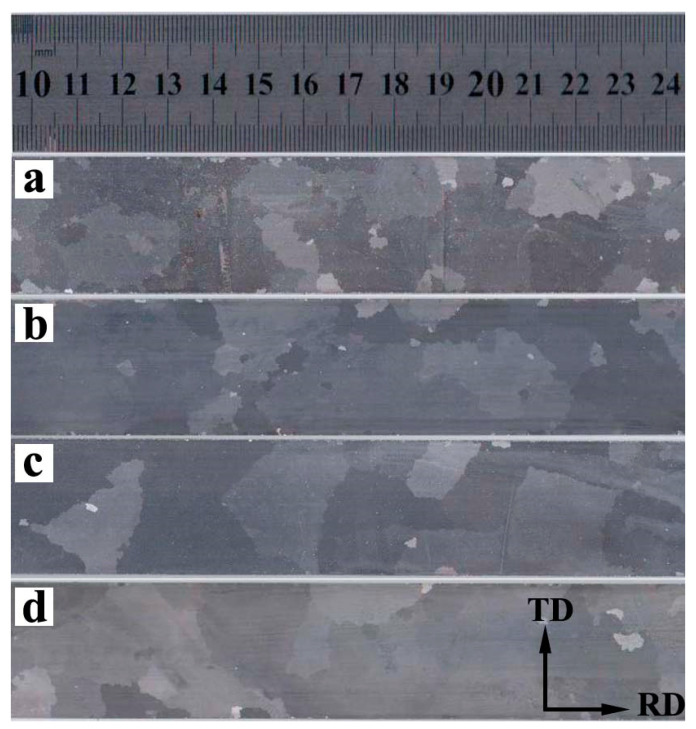
Macrostructure of secondary annealed sheet (**a**) Nb-free, (**b**) Nb-0.005 wt%, (**c**) Nb-0.009 wt% (**d**) Nb-0.025 wt%.

Figure 9 shows the macrostructure of secondary annealed sheet. It can be seen that secondary recrystallization takes place in all the specimens, among which, the grains in S3 show the largest grain size and the best uniformity. Moreover, the grain size reaches the centimeter scale with the maximum size of about 28 mm. There are many small grains in S1, which did not grow up abnormally during final annealing, leading to poor magnetic properties. When the Nb content increases to 0.005 wt%, much more grains grow up abnormally; however, there still exist some small grains after final annealing. Comparing the grains in S3 and S4, the uniform of macrostructure gets worse when the Nb content increases from 0.009 wt% to 0.025 wt%. The corresponding magnetic properties of secondary annealed sheet are shown in Table 3. The best magnetic property is obtained in S3, and *B*_800_ = 1.872 T, *P*_1.7/50_ = 1.25 W/kg, which is consistent with Figure 9. It is well known that the abnormal growth of Goss grain is closely related to its initial environment during final annealing. The growth rate of grains can be calculated by Equation (2) [22]

(2) $$\frac{dR}{dt}=\alpha M\sigma (\frac{1}{R_{c}}-\frac{1}{R}\pm \frac{gZ}{\alpha })$$

where *α* is a constant, *M* is grain boundary mobility, *σ* is grain energy, *R_c_* is the critical size of matrix microstructure, *R* is the size of secondary recrystallized grain, *g* is shape factor, *Z* is pinning resistance. Assuming that the *M* and *σ* are constant and ignoring the pinning effect, it can be concluded that the growth rate increases with the decreases of primary grain size. In addition, the precipitates prevent the normal growth of the primary recrystallized grains and promotes the abnormal growth of Goss grains during secondary recrystallization annealing. According to Section 3.1, the precipitates in nitrided sheet of S3 are the finest and the most dispersed, which would contribute to the complete abnormal grain growth during final annealing. Considering the precipitates microstructure and texture in primary recrystallized sheet, the S3 possesses the finest and the most dispersed precipitates, finest microstructure, and the largest volume fraction of favorable texture. For all these reasons, the grains in S3 could grow up abnormally during final annealing, contributing to the best magnetic properties in S3. Compared to conventional grain-oriented electrical steel [19], the S3 shows better magnetic properties, which further indicates that Nb can be added into silicon steel with a proper content, meanwhile, the reheating temperature can be greatly reduced to 1180 °C.

## 4. Conclusions

In this study, four grain-oriented silicon steels with different Nb content (0–0.025 wt%) were prepared, the effect of Nb content on precipitates, microstructure, texture and magnetic properties of primary recrystallized sheet were studied in detail, the main conclusions are summarized as follows:

(1) The precipitates can be refined by niobium addition, and the finest and the most dispersed precipitates were obtained in steel with 0.009 wt% Nb addition.
(2) The finest primary recrystallized microstructure is obtained in steel with 0.009 wt% Nb due to the strongest pinning force, and the primary recrystallized microstructure changes little when the Nb content is higher than 0.009 wt%.
(3) Adding niobium is beneficial to obtain large volume fraction favorable texture for grain-oriented silicon steel, and the effect of Nb addition is not obvious when the content is higher than 0.009 wt%.
(4) After final annealing, a macrostructure consisting of entirely large grains is obtained in steel with 0.009 wt% Nb, the magnetic induction *B*_800_ and core loss *P*_1.7/50_ are 1.872 T and 1.25 W/kg, respectively.

## Figures and Tables

**Table 1:** Chemical compositions of tested silicon steel (wt%).

Sample	C	Si	Mn	S	Al_s_	Nb	N	Fe
S1	0.056	3.14	0.110	0.0068	0.024	-	0.0076	Balance
S2	0.056	3.11	0.092	0.0064	0.026	0.0050	0.0083	Balance
S3	0.057	3.13	0.091	0.0079	0.025	0.0090	0.0082	Balance
S4	0.060	3.23	0.110	0.0064	0.026	0.0250	0.0075	Balance

**Table 2:** Volume fraction of main texture in primary recrystallized sheet. (Deviation angle < 15°).

Main Textures	Volume Fraction in Different Specimens, %			
	S1	S2	S3	S4
{110}<001>	1.94	2.39	3.24	2.46
{110}<112>	3.39	2.44	2.06	1.55
{111}<110>	2.85	2.36	2.21	1.41
{111}<112>	4.00	5.16	6.61	3.88
{114}<481>	16.5	19.8	23.7	27.7

**Table 3:** Magnetic properties of samples after final annealing.

Sample	Magnetic Induction *B*_800_ (T)	Core Loss *P*_1.7/50_ (W/kg)
S1	1.715 ± 0.003	1.68 ± 0.01
S2	1.806 ± 0.003	1.39 ± 0.01
S3	1.872 ± 0.003	1.25 ± 0.01
S4	1.834 ± 0.003	1.34 ± 0.01
C-GOES [19]	1.84	1.5
